# Mechanism and Potential of Egg Consumption and Egg Bioactive Components on Type-2 Diabetes

**DOI:** 10.3390/nu11020357

**Published:** 2019-02-08

**Authors:** Xiaofeng Wang, Myoungjin Son, Chalamaiah Meram, Jianping Wu

**Affiliations:** 1 Department of Agricultural, Food and Nutritional Science, University of Alberta, Edmonton, AB T6G 2R3, Canada; xwang15@ualberta.ca (X.W.); myoungji@ualberta.ca (M.S.); meram@ualberta.ca (C.M.); 2 Cardiovascular Research Centre, University of Alberta, Edmonton, AB T6G 2R3, Canada

**Keywords:** type-2 diabetes, egg consumption, egg peptides, egg components, insulin

## Abstract

Type-2 diabetes (T2D) is one of the major global health challenges and a substantial economic burden. Egg and egg-derived components have been indicated to possess antioxidant, anti-inflammatory, anti-hypertensive, immunomodulatory, and anti-cancer activities. However, the scientific evidence about the benefits of egg on T2D is debatable. The relationship between egg consumption and the risk of T2D from observational epidemiological studies is not consistent. Interventional clinical studies, however, provide promising evidence that egg consumption ameliorates the risk of T2D. Current research progress also indicates that some egg components and egg-derived peptides might be beneficial in the context of T2D, in terms of insulin secretion and sensitivity, oxidative stress, and inflammation, suggesting possible application on T2D management. The current review summarizes recent clinical investigations related to the influence of egg consumption on T2D risk and in vivo and in vitro studies on the effect and mechanism of egg components and egg-derived peptides on T2D.

## 1. Introduction

Diabetes is a rapidly growing public health problem worldwide, which is characterized by prolonged hyperglycemia and impaired insulin secretion together with or without insulin resistance. Type-1 diabetes is caused by the cell-mediated autoimmune destruction of pancreatic β-cells and accounts for 5–10% of diabetic cases. The majority of diabetes victims (90–95%) are affected by type-2 diabetes (T2D), marked by insulin resistance and relative insulin deficiency [1]. T2D is closely associated with life style—especially diet and exercise—and is preventable. 

According to the World Health Organization, between 1980 and 2014, the global prevalence of diabetes among adults has been increasing from 4.7% (108 million people) to 8.5% (422 million people) [2], in which the westernized lifestyle with dietary changes and lack of exercise is believed to play a role [3,4,5]. Thus, the identification of effective dietary components that can reduce the risk of T2D or slow down the progression of complications is important to improve the quality of life for diabetic patients and people at risk of T2D. Egg is one of the major protein sources in the diet. Also, egg is composed of a broad range of health beneficial components including amino acids, vitamins, minerals, and carotenoids [6]. To date, there are some evidence showing the beneficial property of some egg components and egg-derived peptides in the context of T2D, which are often associated with their anti-oxidative and anti-inflammatory properties [6,7,8]. In this review, we will provide an update on the mechanism and potential of egg, egg components, and egg-derived peptides on T2D management.

## 2. Egg Consumption and the Risk of T2D

The in vivo study addressing the effect of whole egg consumption on T2D is very limited. In Zucker diabetic fatty rats, eight-week feeding of dried whole egg-based diet was shown to reduce blood glucose and triglyceride concentrations, decrease the percentage of body fat, suppress weight gain, and increase circulating 25-hydroxycholecalciferol level, compared to diabetic rats fed with casein-based diet [9,10].

In terms of human study, contradicting observational evidence has been gained from prospective cohort [11,12,13,14,15,16,17,18,19,20,21,22,23], case-control [24], and systematic studies [25,26,27,28,29] in different populations on the relationship between the consumption of egg and T2D risk. A positive association between egg intake and the risk of T2D seemed to be only reported in the US population [12,17,23,29,30,31]. For example, Djousse et al. showed in a 20-year cohort study with 22,071 male physicians (≥40 years at entry) and 39,876 female healthcare workers (≥45 years at entry) in the US that the consumption of 7 eggs/week increased the risk of T2D in both men (Hazard Ratio, HR, 1.58, CI 1.25–2.01) and women (HR, 1.77, CI 1.28–2.43), compared to subjects who had egg consumption of <1/week [12]. In another large cohort study, a positive association between the frequency of egg consumption and the incidence of T2D was reported in 4568 African American subjects (average age: 55 ± 13 years, 64% female) [17]. In addition, higher egg intake was suggested to be correlated with higher blood glucose concentration and negative cardiovascular outcomes in T2D patients in a cohort study conducted in the United Kingdom [32]. However, it has been shown in a prospective cohort study performed in eastern Finland that there was a negative association between egg consumption and the risk of T2D in middle-aged and older men [18,33]. An inverse association was also reported between egg intake and fasting plasma glucose and serum C-reactive protein concentrations [18]. In a Korean study that recruited 7002 middle-aged and older individuals (3318 men and 3684 women), egg consumption was found to be negatively associated with T2D risk in men, but not in women, suggesting the existence of gender differences [34]. Taken together, a meta-analysis that included above mentioned five cohort studies conducted in the US and Finland indicated that egg intake is not a risk factor for T2D [35]. 

The reported discrepancies among observational studies conducted in the US and other countries on the relationship between egg consumption and the risk of T2D could be caused by a number of reasons. In a meta-analysis of prospective cohort studies, Wallin et al. [29] suggested that the various food consumption habits among different countries and cultures could partly explain the heterogeneity in various reports. It has been widely reported that obesity and T2D is correlated with high intake of red meat, fat, and sugar in the diet [36,37], which is characterized as a common dietary pattern in the US population [38]. In fact, Sabate et al. [22] found that there was a significant interaction between meat and egg intake in a cohort study conducted with 55,851 participants in the US. Furthermore, the impact of socioeconomic factors including political environment, culture, income and stress on the prevalence of T2D might contribute to the inconsistency of the results among different countries [39,40]. It was well-established that social determinants such as income, education, housing, and accessibility to nutritious foods are critical players in the development of T2D [41,42]. In addition, alcohol intake and the use of tobacco and drugs are risk factors of T2D [43,44]. In particular, chronic stress, which leads to increases in blood pressure, blood glucose concentration and cortisol level as a consequence of allostatic overload [45], has been closely associated with the increased risk of T2D [46]. Other limitations of the observational studies are the unexcluded environmental factors and selection bias, and the uncontrolled confounding variables [47]. Also, the cooking methods—such as frying, boiling, and steaming—may impact overall nutritional profile and metabolism of egg intake [48,49]. Although cholesterol was once hypothesized as a risk factor for cardiovascular disease, accumulating evidence revoke the hypothesis [27,30,50]; the 2015–2020 Dietary Guidelines for Americans thus removed the limit of cholesterol intake. Currently, there is no consensus on the recommendation of egg intake for T2D patients, however, one egg a day is believed to be safe [51].

Unlike the observational studies, the short-term clinical studies have shown that high egg consumption is correlated with significant improvements on blood lipid level, cholesterol profile, insulin sensitivity, or hyperglycemia [52,53], suggesting higher egg consumption might be beneficial on insulin resistance in T2D. The effect of high-egg consumption on insulin resistance, glucose metabolism, and cholesterol level has been investigated in interventional studies such as randomized clinical trials [52,53,54] and the results were summarized in Table 1. Metabolic syndrome characterized by obesity, hyperlipidemia, hypertension, insulin resistance, and chronic inflammation is believed to be a predictor of T2D [55,56]. To investigate the effects of daily egg intake on lipoprotein metabolism and insulin resistance along with carbohydrate restriction in subjects with metabolic syndrome, Blesso et al. [52] conducted a randomized, single-blind, parallel designed study for 12 weeks to compare the meals containing three whole eggs or yolk-free egg substitute. Although both whole egg group and egg substitute group significantly resulted in improvement on very-low-density lipoprotein (VLDL) particle size, atherogenic lipoprotein subclasses, and oxidized low-density lipoprotein (LDL), whole egg group was shown to have greater increase in high-density lipoprotein cholesterol (HDL-C) and large HDL particles and higher reduction in total VLDL and medium VLDL particles than egg substitute group. Furthermore, plasma insulin concentration and insulin resistance were significantly reduced in whole egg group only [52], Which suggests that egg diet might be effective in managing metabolic syndrome. In addition, the inclusion of whole eggs provided additional benefits in improving insulin resistance compared to yolk-free egg diet [52], indicating the contribution of egg yolk components to this effect. Similarly, 65 subjects with T2D or impaired glucose tolerance were randomized to receive two low-energy diets, one with high protein and high cholesterol content plus two eggs per day, another is high protein-low cholesterol diet containing 100g of lean animal protein. After 12 weeks, diet containing 2 eggs/day did not change the blood lipid profiles of T2D subjects, while HDL-C was improved [53]. In another study conducted by Ratliff et al. in healthy men, one week of daily consumption of eggs for breakfast resulted in reduced energy intake and lower plasma glucose and insulin concentrations compared to a bagel breakfast [57]. A similar result was reported in a randomized, single-blind, crossover trial (*n* = 34) in adults with T2D, which showed that the intake of 2 eggs/day for 12 weeks significantly reduced body weight, waist circumference, visceral fat rating, and percent body fat [58]. The inclusion of eggs in the diet did not change the glycemic hemoglobin A1c (HbA1c) and homeostasis model assessment-insulin resistance (HOMA-IR). However, the exclusion of eggs significantly increased insulin resistance [58]. Also, the significant changes in weight and lipid metabolism suggest that egg might be beneficial in the context of metabolic syndrome [58]. On the contrary, several other clinical trials indicate that egg consumption may not alter metabolic biomarkers associated with T2D [54,59]. Fuller et al. reported that the intake of ≥12 eggs/week for 3 months did not significantly change glycemic response and blood concentrations of total cholesterol, HDL-C, LDL-C, and total triglycerides compared to low-egg diet group (<2eggs/week) in subjects with prediabetes or T2D [54]. This study was revalidated in a longer term (12 months) by the same research group [59]. There was no significant difference between high-egg (≥12 eggs/week) and low-egg diet (<2 eggs/week) in plasma glucose, glycated hemoglobin, 1,5-anhydroglucitol, serum lipids, inflammatory cytokines, oxidative stress, and adiponectin for 12 months [59]. However, the decrease in plasma glucose concentration and HbA1c were higher in high-egg group compared to low-egg group without statistical significance, which is probably due to the mixed subjects with prediabetes and T2D and the lack of a control group who does not consume an egg diet. Although controversial data exists, the current clinical studies provide promising evidence that egg diets ameliorate the risk of T2D. Since the current studies were mostly conducted for short term duration, further studies investigating prolonged consumption of eggs are warranted in individuals with insulin resistance or T2D. 

## 3. Egg Components and T2D

### 3.1. Egg White Hydrolysate (EWH)

EWH produced using alcalase, flavourzyme, neutrase, trypsin, pepsin, pancreatin, and peptidase all showed in vitro activities against oxidative stress and inflammation [61], which are closely inter-connected processes involved in the onset and development of T2D and the progression of complications [62]. Oxidative stress is the result of the overproduction of intracellular reactive oxygen species (ROS) and reactive nitrogen species that damage lipids, proteins, and DNA [63]. The exposure of high concentration of glucose (i.e., 30 mM) causes ROS production through mitochondria pathway in pancreatic β-cells, which in turn inhibit glucose-stimulated insulin secretion (GSIS) [64,65]. Glucose also induces ROS generation and apoptosis in podocytes, whose degeneration predicts nephropathy in the context of T2D [66]. ROS was shown to induce insulin resistance in adipose tissue, skeletal muscle, and hepatocytes by impairing insulin signaling [67,68,69]. Furthermore, there is an association between oxidative stress and negative prognosis of diabetic retinopathy in both animal model and human patients [70]. Prolonged oxidative stress leads to chronic inflammation, while inflammation can induce oxidative stress [71]. Inflammation is the body’s protective response in the purpose of fixing an injury, infection, or irritation, which requires adequate balance between a broad spectrum of mediators, including vasoactive amines, complements, cytokines, chemokines, and eicosanoids [72]. The blood levels of pro-inflammatory cytokines and chemokines such as interleukin (IL)-1, IL-6, IL-18, tumor necrosis factor (TNF)-α, and monocyte chemotactic protein (MCP)-1 were reported to be elevated in T2D patients compared to normal subjects [73,74,75]. Adipose tissue inflammation is believed to play a crucial role in the development of impaired insulin secretion and sensitivity in T2D [76]. TNF-α and IL-1β are known to trigger apoptosis of islets [77], suppress GSIS in both islets and pancreatic beta-cells [78,79], and inhibit insulin signaling in adipose, muscle and liver cells and tissues mainly by suppressing the phosphorylation of insulin receptor, insulin receptor substrate (IRS)-1 and Akt (protein kinase B) [80,81,82,83,84]. Higher egg consumption is associated with lower concentrations of blood pro-inflammatory cytokines in T2D patients [53,85], although egg contains both anti- and pro-inflammatory components, such as anti-inflammatory lutein and zeaxanthin, pro-inflammatory cholesterol, and phospholipids being both anti- and pro-inflammatory [6]. Egg proteins, which are mostly in egg white, are generally considered to be anti-inflammatory, unless inducing an allergic reaction [6].

EWH was also reported to have bile acid-binding, angiotensin I-converting enzyme (ACE)-inhibitory and dipeptidyl peptidase 4 (DPP-4)-inhibitory activities in different potency based on in vitro experiments [61]. ACE is an enzyme that hydrolyzes angiotensin I to produce angiotensin II, which elevates blood pressure, promotes inflammation, and plays a role in the development of insulin resistance [86,87]. DPP-4 is an enzyme degrading incretin hormones such as glucagon-like peptide 1 (GLP-1) [88], which is secreted by intestinal L-cells in response to nutrient load and is known to promote insulin secretion from pancreatic β-cells, preserve β-cell proliferation and regeneration, and inhibit glucagon production [89]. 

There are some in vivo studies indicating that EWH might be beneficial in the context of T2D (Table 2). In spontaneous T2D mice with moderate obesity, eight weeks feeding of protease-produced EWH resulted in an improved glucose tolerance as shown in oral glucose tolerance test and intraperitoneal glucose tolerance test, which was accompanied with a lower plasma insulin concentration, suggesting both insulin secretion and sensitivity could be altered [90]. In high-fat diet (HFD)-fed rats, oral administration of pepsin-prepared EWH for 6 weeks was shown to decrease fat pad mass, increase lean mass, and alleviate glucose intolerance and insulin resistance, which were accompanied with enhanced Akt phosphorylation in liver, muscle, and fat tissues [91]. The plasma concentrations of IL-1α, IL-β, and MCP-1 in HFD-fed rats were also reduced by EWH supplementation [91]. EWH obtained from protease digestion significantly improved the fasting blood glucose concentration and HOMA-IR in non-obese spontaneous diabetic rats, without altering serum levels of insulin and adiponectin, DPP-4 activity, and homeostasis model assessments-insulin secretion [92]. However, in rats fed with a high-fat and high-sucrose diet, the same EWH had no effect on serum glucose and insulin concentrations [93]. Feeding with alcalase-produced EWH for 15 weeks was reported to protect against renovascular damage in obese T2D rats, which was accompanied with suppressed renal mRNA expression of TNF-α, without altering blood GLP-1 and glucose concentrations [94], suggesting the effect may not be dependent on its DPP-4 inhibiting activity. These results indicate that the effect of EWH on glucose and insulin metabolism and T2D-associated inflammation may be dependent on the preparation methods and the obese condition associated with diabetes. However, it should be noted that there is big variation in EWH dose (highest/lowest = ~10) in the current available studies [90,91,92,93,94]. 

The mechanism of EWH’s benefit on T2D is largely unknown. However, there are some in vitro studies supporting the insulin mimetic or sensitizing effects of EWH. Adipose tissue dysfunction plays a critical role in the development of insulin resistance and impaired metabolic homeostasis in T2D [95]. The differentiation of 3T3-F442A mouse pre-adipocytes was shown to be promoted by EWH (prepared by thermoase and pepsin) as evidenced by upregulated lipid accumulation and adiponectin production, possibly by upregulating the protein expression of peroxisome proliferator associated receptor gamma (PPAR)-γ and CCAAT/enhancer-binding protein alpha [96]. EWH treatment also increased the phosphorylation of extracellular signal regulated kinase 1/2 and attenuated c-Jun phosphorylation in 3T3-F442A cells, which was associated with decreased COX-2 expression, a critical regulator of inflammatory pathway. In addition, EWH treatment potentiated Akt phosphorylation induced by insulin [96]. In rat skeletal muscle cells L6, EWH were reported to improve TNF-α-impaired glucose uptake in response to insulin by promoting insulin signaling [97]. 

### 3.2. Lutein and Zeaxanthin

Lutein and zeaxanthin are carotenoid with similar structures to pre-vitamin A (β-carotene), which are concentrated at the macula as the primary pigment molecules [98]. Carotenoids are known for their potent antioxidant activity [99]. Egg yolk contains high level of lutein and zeaxanthin (~143 and 94 μg/yolk, respectively) [100], higher than most fruit and vegetables [101]. The serum concentrations of lutein and zeaxanthin increased 26 and 38% respectively after consuming 1 egg/day for 5 weeks in individuals aged >60y, without changes on concentrations of total cholesterol, LDL-C, HDL-C, and triglyceride in serum [100]. 

Lutein and zeaxanthin function as the major pigment molecules in retina [98]. The macular pigment optical density has been shown to be significantly lower in T2D patients than both type-1 diabetes subjects and normal individuals, although the level was comparable between T2D patients with or without retinopathy [102]. The lutein and zeaxanthin blood concentration in T2D patients with retinopathy was significantly lower than normal subjects [103]. In addition, in a cross-sectional study that involved 111 T2D patients, the plasma concentration of non-pro-vitamin A carotenoids, including lycopene, lutein, and zeaxanthin, was found to be significantly lower in patients with retinopathy than subjects without retinopathy. Furthermore, the ratio of carotenoids that are not pro-vitamin A to the ones that are pro-vitamin A in plasma was negatively associated with the risk of diabetic retinopathy [104].

There are some in vivo studies indicating possible benefits of lutein on insulin resistance and secretion. In rats fed with high-fat diet, lutein administration by gavage for 45 days attenuated hepatic insulin resistance, possibly by increasing the expression of IRS-2, phosphatidylinositol 3-kinase (PI3K), and glucose transporter (GLUT)-4 in liver at both transcription and translation levels [105]. The expression of PPAR-α and sirtuin 1 were also increased, which are players in insulin signaling as well [105]. Lutein and zeaxanthin treatment for eight weeks significantly reduced serum insulin concentration in high-fat diet-fed rats [106], indicating the modulating effect on insulin secretion. 

The possible protecting role of lutein and zeaxanthin against diabetic retinopathy has been suggested by both animal and human studies. Lutein and zeaxanthin were shown to attenuate the oxidative damage of retina in high-fat diet-fed rats which develop obesity and insulin resistance, as evidenced by reduced malondialdehyde concentration and increased activity of antioxidant enzymes in retina [106]. Lutein treatment improved the result of electroretinogram test, reduced oxidative stress, and suppressed nuclear factor kappa B (NF-κB) activity in retina of diabetic rats induced by alloxan [107], which is a toxic glucose analogues causing β-cell damage through generating free radicals [108]. Zeaxanthin was reported to have similar protective property on the development of diabetic retinopathy as well. In diabetic rats induced by streptozotocin—which is another β-cell damaging glucose analogue mainly acting by inducing DNA damage [108]—zeaxanthin supplementation inhibited the levels of lipid peroxide, oxidatively modified DNA, electron transport complex III, nitrotyrosine, and mitochondrial superoxide dismutase in the retina, which were accompanied with reduced retinal expression of vascular endothelial growth factor and intercellular adhesion molecule-1 [109]. In db/db diabetic mice, dietary supplementation of wolfberry which has high lutein and zeaxanthin content was reported to restore the thinned retina, especially the inner nuclear and photoreceptor layers, and protect the integrity of the retinal pigment epithelia by improving mitochondria function and attenuating hypoxia, oxidative stress, and ER dysfunction [110,111]. The protecting effect of lutein on retina in the context of T2D was shown to be comparable with docosahexaenoic acid in terms of oxidative stress, apoptosis, thickness of the outer and inner nuclear layers, and electroretinogram in diabetic rats [112]. In T2D patients with diabetic retinopathy, three-month supplementation of lutein and zeaxanthin significantly improved visual acuity, increased contrast sensitivity, and decreased fovea thickness [103]. These evidence suggests that dietary lutein and zeaxanthin supplementation might be a promising strategy to alleviate the development of diabetic retinopathy, which requires further investigation. 

### 3.3. Choline

Choline is a serine-derived water-soluble amine and is the build block of phosphatidylcholine and sphingomyelin, which are essential membrane phospholipid and precursors of second messengers diacylglycerol and ceramide. Choline is also a precursor of an crucial neurotransmitter acetylcholine, which is involved in voluntary muscle movement and cognitive function [113]. Another important physiological function of choline is that it is required for the transport of triglyceride in lipoprotein from liver [114]. 

Eggs, liver, and peanuts are good food sources of choline. The choline content in whole egg is ~100 mg/egg, mainly in egg yolk [115,116]. There are some evidence showing the beneficial effect of choline on glucose and insulin metabolism. In mice with metabolic syndrome, muscle synthesis of fatty acid and triglyceride was reduced by choline dietary supplementation, whereas glycogen generation was increased. In addition, phosphorylation of IRS-1 and Akt in muscle was enhanced by choline [117]. In a study that involved 2394 adults from Newfoundland, the dietary choline intake was negatively associated with blood concentrations of fasting glucose and insulin and HOMA-IR in both males and females with age, total calorie intake, and physical activity level being controlled [118]. Choline was also reported to aid in the delivery of insulin ingested orally. An ionic liquid consist of choline and geranate was shown to improve oral insulin delivery and result in a significantly reduced blood glucose level for up to 12 hours in rats [119], which were attributed to enhanced paracellular transport and reduced enzymatic degradation of insulin. 

However, there are also studies showing contradictory results. Phosphatidylethanolamine N-methyltransferase-deficient mice, which display impaired choline de novo synthesis, were protected from high-fat diet-induced obesity and insulin resistance [120]. In addition, a choline-deficient diet led to an improved glucose tolerance and less weight gain in wild-type mice [120]. Similar attenuation on weight gain and improvement on glucose tolerance and insulin resistance by a choline-deficient diet were seen in both ob/ob obese mice and high-fat diet-fed wild-type mice [121]. In a human study that involved three prospective cohorts with 203,308 subjects (both male and female) without diabetes, cardiovascular disease, and cancer [122], a valid food-frequency questionnaire was used to assess the dietary intake of phosphatidylcholine, which can be degraded to choline by gut microbes. The results showed that higher phosphatidylcholine intake was associated with increased risk of T2D [122]. 

## 4. Egg-Derived Peptides and T2D

### 4.1. Alpha-Glucosidase Inhibitory Peptides

Although individual variation and abdominal discomfort have been reported, synthetic α-glucosidase inhibitors, such as acarbose and voglibose, were shown to be beneficial against postprandial hyperglycemia in diabetic individuals by inhibiting α-glucosidase, which hydrolyzes carbohydrate and release monosaccharides for absorption in the small intestine [123,124]. Both acarbose and voglibose were effective on improving glucose turnover in T2D patients who were taking insulin and metformin and blood glucose levels were not adequately controlled [125], indicating α-glucosidase inhibitors could be valuable supplements for T2D patients. It was also reported that voglibose diet supplementation augmented GLP-1 secretion in both healthy people and mice with T2D, which is attributed to the delaying effect on carbohydrate absorption [126,127]. However, acarbose was not shown to increase GLP-1 secretion in patients with T2D [128], indicating α-glucosidase inhibitors may have distinct potency on promoting GLP-1 secretion in different health conditions. Thus, food-derived α-glucosidase inhibitors might be attractive options replacing synthetic ones for the management of T2D. Eight peptides with α-glucosidase inhibitory activity have been identified in egg albumin with peptide KLPGF being the most potent one, which was shown to have comparable potency as acarbose [129]. Another egg white derived peptide RVPSLM is ~3-fold more potent than acarbose in glucosidase inhibition [130]. Peptide VTGRFAGHPAAQ with high α-glucosidase inhibitory activity was identified from egg yolk protein [131]. However, effect of α-glucosidase inhibitory peptides from egg on glucose and insulin metabolism in the context of T2D requires further study. 

### 4.2. ACE Inhibitory Peptides

In the context of T2D, angiotensin II, produced from angiotensin I by ACE, has been implicated to play a role in the development of insulin resistance. Angiotensin II was reported to inhibit insulin/ PI3K/Akt signaling, induce oxidative stress by activating NADPH oxidases, and upregulate inflammation by activation of NF-κB [86], which is known to initiate the transcription of pro-inflammatory genes including cytokines, chemokines, and adhesion molecules [132]. The action of angiotensin II is believed to be mainly through a G-protein coupled receptor, angiotensin II type 1 receptor [133]. ACE inhibitors, traditionally used for anti-hypertension purpose, have been reported to exert favorable effects on kidney, heart and eye functions in T2D patients [134].

Milk derived ACE inhibitory tripeptides, IPP (Ile-Pro-Pro) and VPP (Val-Pro-Pro), induced similar adipogenic differentiation to insulin, which was accompanied with restored adipokine levels and reduced activation of NF-κB [135]. An ACE inhibitory tripeptide, IRW (Ile-Arg-Trp), which is derived from egg white ovotransferrin, has been indicated to be beneficial against insulin resistance. In fully differentiated L6 myoblasts, IRW were reported to restore TNF-α-impaired insulin- stimulated glucose uptake by promoting phosphorylation of IRS-1 tyrosine residue and Akt, which were accompanied with decreased phosphorylation of p38 and c-Jun N-terminal kinases [97]. IRW in vitro treatment also reversed angiotensin II-impaired insulin-stimulated glucose uptake in L6 myoblasts, by reducing serine phosphorylation of IRS-1, increasing Akt phosphorylation, upregulating GLUT-4 translocation, decreasing expression of angiotensin II type 1 receptor, and inhibiting ROS generation [136]. The effect and mechanisms of ACE inhibitory peptides from eggs on diabetes largely remain to be elucidated. 

### 4.3. DPP-4 Inhibitory Peptides

Several DPP-4 inhibitors (also known as gliptins) have been approved in the US, Europe, Japan, and South Korea to treat T2D, which are supposed to augment the bioavailability of incretin hormones, prolong the action of insulin, and thus provide benefits on postprandial glucose response [137]. Peptide LPQNIPPL originated from water-soluble extract of a gouda-type cheese was reported to have DPP-4 inhibitory activities in vitro, and improve glucose tolerance as shown in oral glucose tolerance test in healthy rats when administrated together with glucose by intraperitoneal injection [138]. In addition, oral administration of peptides with DPP-4 inhibitory activity derived from the porcine skin gelatin hydrolysates were demonstrated to improve glucose tolerance in diabetic rats in 21 and 42 days after streptozotocin injection [139]. Three peptides, YINQMPQKSREA, VTGRFAGHPAAQ, and YINQMPQKSRE, with DPP-4 inhibitory activity have been identified in egg yolk protein, with YINQMPQKSRE being the most active one (IC50 = 222.8 µg/mL) [131]. However, further studies elucidating the effect of egg-derived DPP-4 inhibitory peptides in the context of T2D are needed. 

## 5. Concluding Remark

To date, the observational epidemiological evidence about the egg consumption and the risk of T2D is not consistent, which might be the result of different dietary pattern and socioeconomic factors. However, it has been indicated that there is association between higher egg consumption and improved blood lipid profile, insulin sensitivity, and glucose response in interventional clinical trials.

**Figure 1:**
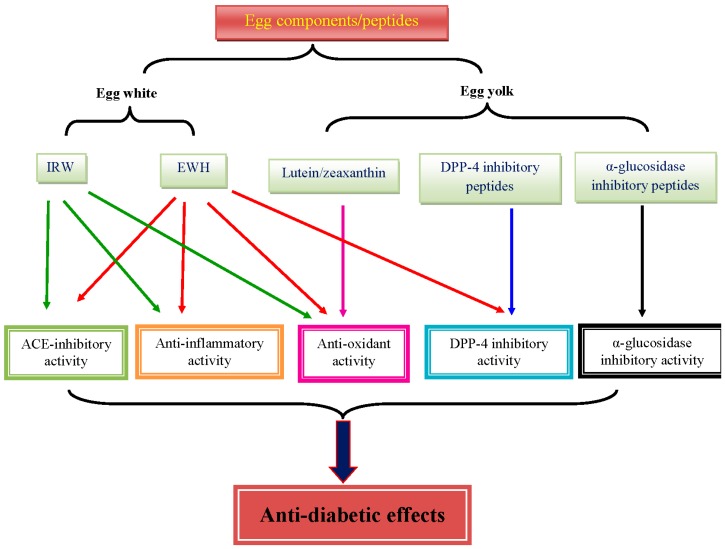
Major bioactivities of egg components and egg-derived peptides that possibly contribute to the benefits in T2D.

EWH, lutein, zeaxanthin, and ACE inhibitory tripeptides from egg have been shown to have some benefits against glucose and insulin intolerance, oxidative stress, and inflammation in the context of T2D (Figure 1). The effect of EWH seems to be related to the production process, which needs optimization. In addition, the role of α-glucosidase inhibitory peptides, DPP-4 inhibitory peptides, and choline from egg in T2D is poorly understood. Thus, more mechanistic studies are warranted to elucidate the role of each components of egg on T2D. Furthermore, the absorption and bioavailability of the egg components and egg-derived peptides largely remain to be addressed.

## Figures and Tables

**Table 1:** Recent clinical studies investigating the effect of egg consumption on T2D

Egg Dose	Subjects	Duration	Study Design	Primary and Secondary Outcomes	Results
3 eggs/day [52]	37 women with metabolic syndrome *; aged 30–70 years	12 weeks	Randomized, single blind, parallel design	Plasma lipids, apolipoprotein, oxLDL, CETP and LCAT	Improved HDL, large HDL particles, total and medium VLDL particles, HOMA-IR, and LCAT activity
2 eggs/day [53]	65 subjects with T2D or impaired glucose tolerance; aged 54 ± 8.2; BMI 34.1 ± 4.8 kg/m^2^	12 weeks	Randomized, controlled, parallel design	Blood lipid, glucose, insulin, HbA1c, CRP and apoprotein-B, homocystein	Increased HDL cholesterol; improved glycemic and lipid profiles
Egg breakfast [57]	21 healthy men; aged 20–70 years	1 week	Randomized, Cross-over	Fasting blood glucose, plasma insulin, ghrelin, leptin, GLP-1, PYY	Less variation in plasma glucose and insulin; reduced ghrelin response and energy intake
2 eggs per day [58]	34 adults with T2D (14 postmenopausal women and 20 men); mean age = 64.5 years	12 weeks	Randomized, controlled, single-blind, cross-over	Glycated hemoglobin, systolic blood pressure, body mass index, visceral fat rating, waist circumference, and percent body fat	Reduced body mass index, visceral fat, waist circumference and percent body fat; unchanged glycemic control
High-egg diet (≥12 eggs/week) or low-egg diet (<2 eggs/week) [54]	140 aged subjects with prediabetes or T2D; BMI ≥ 25 kg/m^2^	3 months	Randomized, controlled, parallel-arm	Plasma blood glucose, HbA1c, TC, LDL-C, HDL-C, TG, apolipoprotein B, CRP	No significant changes between groups
High-egg diet (≥12 eggs/week) or low-egg diet (<2 eggs/week) [59]	128 subjects with prediabetes or T2D; aged ≥18 years; BMI ≥25 kg/m^2^	12 months	Randomized, controlled, parallel-arm	Plasma glucose, HbA1c, 1,5-anhydroglucitol, traditional serum lipids, markers of inflammation, high-sensitivity C-reactive protein, interleukin 6, soluble E-selectin, oxidative stress, and adiponectin	No significant changes between groups

* The National cholesterol Education Program’s Adult Treatment Panel III report definition [60]. cholesteryl ester transfer protein: CETP; C-reactive protein: CRP; glucagon-like peptide 1: GLP-1; lecithin cholesterol acyltransferase: LCAT; total cholesterol: TC; triglyceride: TG; oxidized LDL: oxLDL.

**Table 2:** In vivo studies of the effect of EWH on T2D.

EWH Preparation Method	Animal Model/Group	EWH Dose/ Duration	Major Results
Protease [90]	Nagoya-Shibata-Yasuda mice	27.6% (w/w, diet) /8 weeks	Decreased plasma glucose and insulin concentration; improved insulin resistance
Alcalase [94]	Obese Zucker rats	1 g/kg (body weight)/day/15 weeks	Reduced renal mRNA expression of IL-1β, IL-13, and TNF-α; decreased renal P22(phox)protein expression; unchanged blood GLP-1 and glucose concentration
Thermolysin and Pepsin [91]	High-fat diet-fed rats	4% (w/w, diet)/6 weeks	Reduced plasma IL-1α, IL-β, and MCP-1 concentrations and fat pad mass; increased lean mass and upregulated Akt phosphorylation in liver, muscle, and fat tissues; improved glucose tolerance and insulin sensitivity
Protease [92]	Goto-Kakizaki rats	27.6% (w/w, diet)/6 weeks	Decreased fasting blood glucose concentration and triglyceride content in muscle; improved HOMA-IR;
Protease [93]	Rats fed with a high-fat and high-sucrose diet	39.4% (w/w, diet)/6 weeks	Reduced food intake, body weight gain and fat deposition; decreased stearoyl-CoA desaturase and glucose-6-phosphate dehydrogenase activity in liver and muscle; suppressed serum levels of triacylglycerol and leptin; increased muscle weight; upregulated fecal excretion of triacylglycerol, cholesterol, and total bile acids
